# Water–Air Interface to Mimic In Vitro Tumoral Cell Migration in Complex Micro-Environments

**DOI:** 10.3390/bios12100822

**Published:** 2022-10-03

**Authors:** Martina Conti, Ilaria Bolzan, Simone Dal Zilio, Pietro Parisse, Laura Andolfi, Marco Lazzarino

**Affiliations:** 1 Department of Physics, University of Trieste, 34127 Trieste, Italy; 2 IOM-CNR, Institute of Materials Foundry—National Research Council, 34149 Trieste, Italy

**Keywords:** cell migration, micro-patterned platform, cellular micro-environment

## Abstract

The long-known role of cell migration in physiological and pathological contexts still requires extensive research to be fully understood, mainly because of the intricate interaction between moving cells and their surroundings. While conventional assays fail to capture this complexity, recently developed 3D platforms better reproduce the cellular micro-environment, although often requiring expensive and time-consuming imaging approaches. To overcome these limitations, we developed a novel approach based on 2D micro-patterned substrates, compatible with conventional microscopy analysis and engineered to create micro-gaps with a length of 150 µm and a lateral size increasing from 2 to 8 µm, where a curved water–air interface is created on which cells can adhere, grow, and migrate. The resulting hydrophilic/hydrophobic interfaces, variable surface curvatures, spatial confinements, and size values mimic the complex micro-environment typical of the extracellular matrix in which aggressive cancer cells proliferate and migrate. The new approach was tested with two breast cancer cell lines with different invasive properties. We observed that invasive cells (MDA-MB-231) can align along the pattern and modify both their morphology and their migration rate according to the size of the water meniscus, while non-invasive cells (MCF-7) are only slightly respondent to the surrounding micro-environment. Moreover, the selected pattern highlighted a significative matrix deposition process connected to cell migration. Although requiring further optimizations, this approach represents a promising tool to investigate cell migration in complex environments.

## 1. Introduction

Cell migration is a complex biological process that allows cells to reach a targeted location to perform their functions [1,2]. Although an evolutionarily conserved ability, cell migration occurs through a variety of mechanisms influenced by both intrinsic and extrinsic cues, and relies on an intricate network of cytoskeletal proteins, intercellular signaling pathways, and transmembrane proteins [1,3]. The main source of external signals is the cellular environment, which consists of a dense extracellular matrix (ECM) and other neighboring cells [4] and is physically characterized by stiffness, curvatures, topographies, and the degree of spatial confinement, which can vary locally [5].

The heterogeneous and intricate nature of the migration process makes its study extremely complex. Most migration studies are performed on two-dimensional platforms, such as the wound-healing assay and the fence assay [6,7], that allow for the easy tracking and imaging of cells, but at the cost of an oversimplification of the *in vivo condition*. Several 3D assays, based on gels or spheroids, have been developed as more physiologically relevant models [6,8]. Despite their ability to integrate different types of signals, such as spatial confinement or stiffness gradients, they require advanced imaging techniques [1,8].

The need for a platform that allows for an easy and efficient analysis of cell migration arises from the relevance of this process in many physiological and pathological contexts. Cells migrate to repair injured tissues, properly assemble organs during development, or start the inflammatory response [1,2]. Moreover, cell migration is altered in inflammatory and autoimmune diseases [9], as well as in neurological disorders [10]. Cell migration is also essential for the formation of cancer metastases [11] and its study is extremely relevant for the development of diagnostic [12] and therapeutic [13,14] strategies. Thus, several experimental models have shown that cells move according to stiffness [15] or curvature gradients [16], and can adopt different morphology organization and migration modes depending on the architecture and structural organization of the micro-environment [5,17,18,19,20].

To address some peculiar characteristics of the ECM, i.e., hydrophilic/hydrophobic interfaces, water menisci, and surface curvature, we have developed a novel micro-patterned 2D system that uses the meniscus created at the water–air interface of micro-structured gaps to study the effect of these structures on cancer cell behavior by integrating different microscopy techniques.

The topographic features of the ECM consist of a mixture of fibers, ridges, and sub-micrometer pores [21,22]. It has been shown that the growth and morphology of cells vary as a function of the geometrical features of the substrates. In particular, cells can pass through the pores and channels of the ECM, provided that the diameter of these structures is not smaller than the 10% of the cross-sectional area of their nucleus [5,21]. Cell diffusion through membrane pores with sizes between 3 and 12 µm has also been reported [6].

According to these characteristics, we structured substrates with rectangular micro-gaps of 2, 4, and 8 µm wide and we studied their effects on the morphology and migration behavior of MDA-MB-231 and MCF-7 cancer cells. Moreover, since the fiber-like pathways that cancer cells can create and follow during their migration through the tumor microenvironment range from 100 to 600 µm [5,21], we chose a micro-gap length of 150 µm, which has been commonly adopted in previous studies on the migration of breast cancer cells [23]. Using this length, we created an array composed of 12 micro-gaps for each $Si_{3}N_{4}$ chip, which may offer, in a single experiment, a data set large enough for improved statistical analysis.

Our findings highlight that breast cancer cells with a highly invasive behavior differently perceive the micro-gaps’ size and, as a result, they assume specific morphological, cell orientation, and migratory features. This micro-patterned 2D system represents an interesting approach not only to study cancer cell migration but that, with further optimization, could be extremely useful in tumor diagnosis to evaluate the malignancy grade.

## 2. Materials and Methods

### 2.1. Fabrication of the Substrates

**Figure 1:**
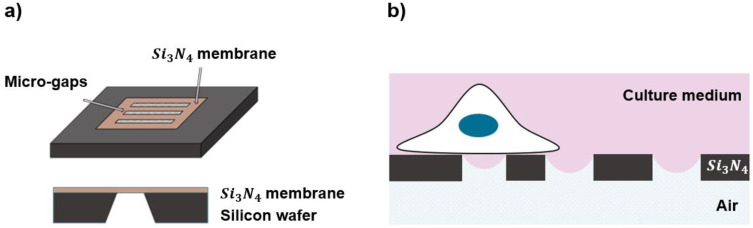
(**a**) Top and side view of the chip with substrate, where the micro-patterned structures were created in the SiN membrane; (**b**) a schematic representation of half-wet configuration experienced by a cell grown on the top of the patterned substrates, where the culture medium is covered on the “cell side” and the substrates are exposed to air on the other side or “air side”.

The substrates consisted of a chip where silicon nitride $(Si_{3}N_{4}$) membranes with a surface micro-structured pattern (Figure 1a) were obtained, combining standard UV lithography and electron beam lithography (EBL) starting from a 500 µm silicon wafer coated on both sides with a 2 μm thick low-stress LPCVD silicon nitride ($Si_{3}N_{4}$) film. At first, a 150 nm thick chromium film was deposited on both sides by DC magnetron sputtering. The $Si_{3}N_{4}$ window pattern and the relative markers for the subsequent EBL process on the cell side were transferred on the air side from two different masks to the substrates using double-sided aligned UV exposure. The chromium layer was wet-etched in a water solution of acetic acid and ceric nitrate ammonium. For clarity, in the following sections, the side of the substrates on which cells will be seeded will be referred to as the “cell side” and the other as the “air side”. The micro-structured pattern was then aligned on the suspended $Si_{3}N_{4}$ membranes and generated on the cell-side of the substrates by EBL. As shown in Figure S1a, the micro-structured pattern includes 4 microarrays, each consisting of 3 rectangular gaps, spaced 50 µm apart, 150 µm long, and with different widths of 2, 4, and 8 µm. Once the pattern was transferred to the substrates, the exposed $Si_{3}N_{4}$ was dry-etched by Reactive Ion Etching (RIE) on the air side and by Inductively Coupled Plasma (ICP-RIE) on the cell side, using a mixture of *SF_6_/C_4_F_8_/O_2_* structures.

Finally, the patterns were released in a solution of *KOH* (30 wt%; 80 °C) to expose the $Si_{3}N_{4}$ surface that was previously adherent to the underlying silicon wafer and the substrates with the final microstructures were characterized by SEM (Figure S1b).

### 2.2. Half-Wetting Assembly

The presence of a water meniscus on the substrates is obtained by maintaining them in a so called “half-wetting condition”, with the cell-side in contact with the culture medium and the other side exposed to air (Figure 1b). The water–air interface across the gaps is required to create the meniscus, which is then used as topographical feature to mimic a confined and curved space. For this purpose, we designed and developed a special assembly (Figure S2) that was compatible with an inverted microscope equipped with a live-cell-imaging setup, with the chips sealed using ReproRubber^®^ ThinPour on a commercial petri dish in which 2 mm diameter holes, exceeding the size of the ${\mathrm{Si}}_{3}N_{4}$ windows, were cut using a CO_2_ laser. Initial attempts to attach the chips to petri dishes without holes resulted in condensation on the bottom of the chips during incubation at 37 °C, which could compromise the desired half-wet configuration for live cell experiments.

To ensure sterility throughout the entirety of each experiment, the petri dish with the holes was covered with a petri cap (to avoid any contaminations during the transfer of the device from incubator to the live-cell-imaging setup) and a PMMA ring (with a diameter of 2 cm and a height of 3 mm) was placed between the cap and the Petri dish with the holes. Once the functionalized substrates were glued to the Petri dish, the components were sterilized with UV exposure for 20 min and then assembled, as shown in Figure S2b.

### 2.3. Functionalization of the Substrates

To enhance cell adhesion, substrates were functionalized with the 3-amino-propyl-triethoxysilane (APTES) using an alternative protocol based on chemical vapor deposition (CVD) process, as described in the literature [24,25]. In detail, the substrates were fixed on a glass substrate with Kapton tape and subjected to a plasma O_2_ treatment (1 min, 40 W, 100 bias) to clean the surface and expose the silanol groups for the subsequent functionalization. A glass Petri dish was filled with 300 µL of ATPES and placed on the bottom of a glass vacuum chamber, maintained at 10^−3^–10^−5^ mBar, and heated to 50 °C. Samples were held upside-down 20 cm above the Petri dish and exposed to the APTES vapor for 4 h at 50 °C in continuum vacuum. Finally, the substrates were cured at 120 °C for two hours in another vacuum oven. Functionalization was verified with contact angle measurements (Figure S3a) and its efficacy for cell-adhesion purposes was assessed by measuring cell density on native and functionalized ${\mathrm{Si}}_{3}N_{4}$ membranes (Figure S3b).

### 2.4. Cell Culture

All the experiments were performed with MDA-MB-231 (ECACC 92020424) and MCF-7 (Sigma-Aldrich, St. Louis, MO, USA, ECACC, #92020424) cells. Cells were cultured in a Dulbecco’s modified Eagle medium (DMEM-High Glucose), with L-glutamine and sodium pyruvate (Sigma-Aldrich, St. Louis, MO, USA), and supplemented with 10% (*v*/*v*) of fetal bovine serum (FBS) (Sigma-Aldrich, St. Louis, MO, USA) and 1% (*v*/*v*) penicillin-streptomycin (EuroClone Spa, Pero (MI), Italy). Both cell lines and cells seeded on the substrates were cultured in the incubator at 37 °C, 5% CO_2_.

### 2.5. Experimental Procedure

**Figure 2:**
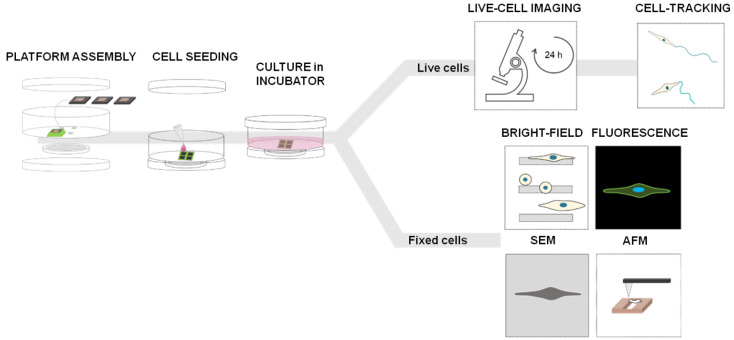
Experimental workflow with the 2D micro-patterned system—4 holes are created on the bottom of a commercial petri dish (35 mm of diameter) using the laser cutter instrument and each chip is fixed on each hole with a biocompatible glue with the top side facing up; cell seeding—a 25 µL drop of cell suspension is placed on the chip surface and incubated for 20 min in incubator (37 °C and 5% CO_2_) to allow cells to adhere, while the bottom side beyond the gap is kept dry; the petri-dish is filled with the medium (2 mL final volume) and the chamber is sealed using a petri dish cap on the bottom to ensure sterile condition; for live cell analysis, samples were left in the incubator (5% CO_2_, 37 °C) for 12 h and then observed with the Live-Cell-Imaging Instrument for the next 12 h; for fixed cell analysis, cells were fixed 24 h after incubation and analyzed using various microscopy techniques.

The experiments with 2D-micropatterned system were performed according to the workflow shown in Figure 2: each chip (dark grey square) with the final microstructures was glued on each hole created in the petri dish and the assembly was mounted; a 25 µL droplet containing about 3.000 cells was placed on the top of each chip under sterile conditions and incubated in a humidified atmosphere at 37 °C and 5% CO_2_ for 20 min to allow the cells to adhere while the bottom was kept dry; the petri dish was filled with the medium (2 mL final volume) and incubated at different times. For live cell imaging, samples were kept in humidified incubator at 37 °C supplemented with 5% CO_2_ for 12 h and then images were recorded by microscope for the next 12 h, always under the same environmental conditions (Figure 2, on top). For brightfield (BF) imaging, scanning electron microscopy (SEM), and atomic force microscopy (AFM) (Figure 2, on bottom), samples were kept in an incubator for 24 h (37 °C and 5% CO_2_) and then fixed in PFA 4%. For fluorescence imaging, fixed samples were stained with phalloidin (Alexa Fluor™ Plus Phalloidin) to visualize actin-cytoskeleton, and DAPI (GeneTex Inc., Irvine, CA, USA, GTX16206) to identify the nuclei.

### 2.6. SEM Imaging

After cell culturing, the medium was removed, and the cells were rinsed with phosphate-buffered saline (PBS). Then, cells were fixed in 4% paraformaldehyde for 20 min at room temperature. After incubation, they were rinsed in PBS and then dehydrated in graded ethanol, dried, and coated with a thin layer of Au/Pd. SEM was performed with the instrument LEO 1540XB (Carl Zeiss AG, Jena, Germany) and images were acquired using secondary electron (SE) signal.

### 2.7. AFM Imaging

AFM images were acquired in AC mode in air by an MFP-3D-Bio instrument (Asylum Research/Oxford Instruments) with silicon cantilevers (NSG01_DLC, NT-MDT, radius of curvature < 5 nm, and spring constant 2 < k < 10 N/m). Large scans (10–20 μm) across the grooves were acquired at 256-pixel resolution and 0.1–0.2 Hz scan rate, while smaller scans (2–4 μm) were acquired at 256-pixel resolution and 0.5–1 Hz scan rate. Image analysis and surface reconstruction to account for double tip effects has been carried out with the software Gwyddion 2.59.

### 2.8. Morphological Analysis

The bright-field images were used to calculate the aspect ratio and orientation angles of the MDA and MCF-7 cells by the free-software Fiji (ImageJ). After fitting the cells with ellipses, the cell orientation was calculated as the angle between the major axis of the ellipse and a reference axis parallel to the long side of the micro-gap structure, while the aspect ratio was obtained by the ratio between the major and minor axis of the ellipse. For the analysis of the cell orientation, we used the absolute value of the angles, which is all that is needed to describe cell orientation, so that the angle values range from 0° to 90°.

### 2.9. Migration and Density Analysis

The analysis of cell migration was performed using the Olympus Scan R 3.1 microscope, equipped with the live-cell-imaging module. Cells were seeded both on substrates with micro-gaps and on flat $Si_{3}N_{4}\;$ membranes, left in the incubator (5% CO_2_, 37 °C) for 12 h to ensure adhesion and cell recovery, and then monitored for the next 12 h inside the chamber of the live-cell-imaging setup.

For each experiment, images were acquired every 15 min. The resulting images were analyzed with ImageJ to obtain the number of cells adhering to the micro-gaps and the velocity of migrating cells. The velocity of migration was obtained using two plug-ins in ImageJ, following the protocol described by Pijuan et al. for single-cell tracking [2]. The “Manual Tracking” plug-in enabled individual cell tracking. The coordinates obtained with this plug-in were entered in the “Chemotaxis and Migration Tool” (Ibidi 2.0, Fiji (ImageJ)) to extract the total distance covered by a cell both on flat substrate and in gaps.

The velocity values were calculated for cells that were moving along and outside the gaps on flat substrate, using the Accumulated Distance and the time intervals, in which trajectories of the cells were followed with the “Manual Tracking” plug-in.

The number of cells adhering to the micro-gaps (cell density) was evaluated as a ratio between the density of cells on the micro-gaps and the density of cells on the surrounding membrane, as described in the Supplementary Materials (Paragraph S4 and Figure S4). In particular, we considered the surrounding membrane as the area in which the cells could come in contact with the gap during the time of observation. Therefore, a ratio larger than one means that there are more cells that become trapped by the gap than those who leave the gap, or in other words, that cells “prefer” the gap area to the surrounding membrane.

### 2.10. Statistical Analysis

Statistical analysis was performed using GraphPad Prism (version 8.4.3). Statistical significance was evaluated by a two-tailed Student’s *t* test, where *p* ≤ 0.05 (*), *p* ≤ 0.01 (**), *p* ≤ 0.001 (***) and *p* ≤ 0.0001 (****) were considered statistically significant.

## 3. Results

### 3.1. Morphological Evaluations

**Figure 3:**
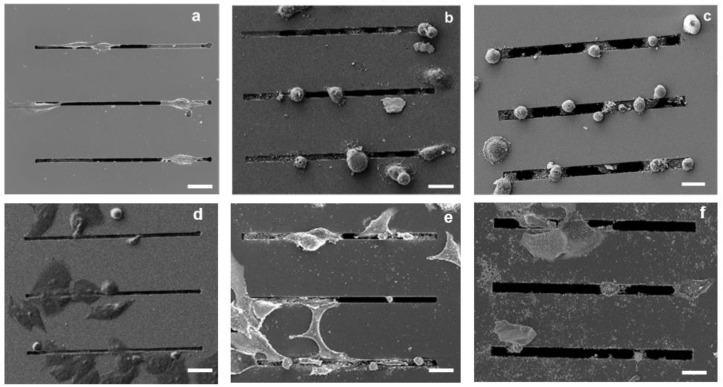
SEM images of MDA-MB-231 cancer cells cultured on microstructures with different gap sizes: 2 μm (**a**), 4 μm (**b**), and 8 μm (**c**); SEM images of MCF-7 cancer on microstructures with different gap sizes: 2 μm (**d**), 4 μm (**e**), and 8 μm (**f**); Scale bars: 25 µm.

Both the SEM images and the fluorescence microscopy analysis performed 24 h after seeding showed qualitatively more distinct morphological features in the MDA-MB-231 cells compared with the MCF-7 cells as a function of gap size. In particular, as shown by the SEM images (Figure 3), the MDA-MB-231 cells on the 2 μm gap appear to have an elongated morphology (Figure 3a), whereas the cells on the 4 and 8 μm gaps appear to have a more rounded morphology (Figure 3b,c). In contrast, as shown in Figure 3d–f, the MCF-7 cells appear to be less responsive to the topography of the patterned substrates, with a minimal elongation along the gaps.

**Figure 4:**
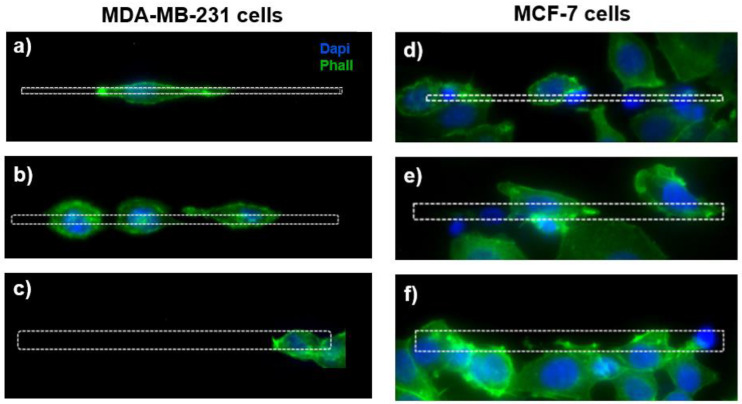
On the left, the fluorescence images of MDA-MB-231 cells grown on micro-gaps with different gap size. Cells on gaps 2 μm wide (**a**) appear more elongated than in gaps 4 μm (**b**) and 8 μm (**c**) wide. On the right, the fluorescence images of MCF-7 cells’ fluorescence images cultured on the 2 µm (**d**), 4 µm (**e**), and 8 µm (**f**) wide structures. Green—actin cytoskeleton (stained with Phalloidin); blue—nuclei (stained with DAPI).

The visualization of the actin cytoskeleton through the fluorescence images shows a different morphology of the MDA-MB-231 cells grown on the microstructures as a function of the micro-gaps’ size (Figure 4a–c). For the MDA-MB-231 cells, the cell elongation decreases with the gap width, and for cells on the 2 µm wide micro-gaps we can observe membrane protrusions inside the gaps and an extended actin cytoskeleton, while the cells on the 8 µm wide micro-gaps exhibit a rounded morphology.

On the contrary, for the MCF-7 cells, the morphology seems to be unaffected by the topography (Figure 4d–f).

**Figure 5:**
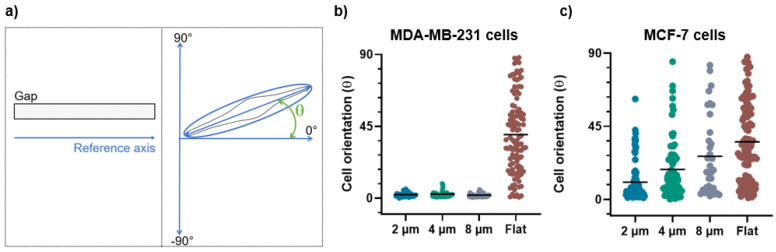
(**a**) The schematic representation of the orientation angle as a function of cells’ positioning along the micro-gaps. After indicating the reference axis as parallel to the major axis of the micro-gap (on left), the cell orientation was calculated as the angle between the major axis of the cell and the reference axis (on right). (**b**) Orientation angles for MDA-MB-231 cells on 2 µm (n = 95), 4 µm (n = 78), and 8 µm (n = 52) wide micro-gaps and on flat membranes (n = 150). (**c**) Orientation angles for MCF-7 cells on 2 µm (n = 94), 4 µm (n = 73), and 8 µm (n = 36) wide micro-gaps and on flat membranes (n = 101). All data plotted are the absolute values of the evaluated angles. The black line is the mean value for each data set.

**Figure 6:**
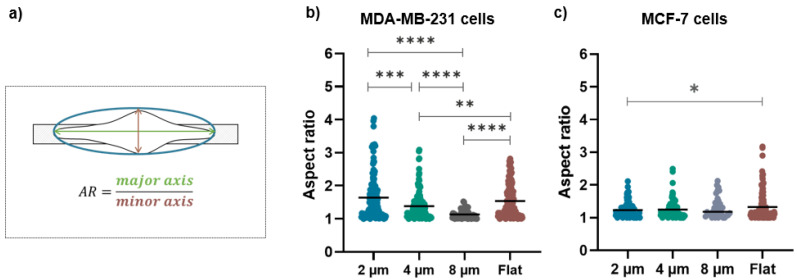
(**a**) The schematic representation of the long and short axes of cells on structures with different gap sizes used to evaluate the aspect ratio (AR); (**b**) aspect ratio values for MDA-MB-231 cells on 2 µm (n = 146), 4 µm (n = 132), and 8 µm (n = 61) wide micro-gaps and on flat membranes (n = 150); (**c**) aspect ratio values for MCF-7 cells on 2 µm (n = 108), 4 µm (n = 94), and 8 µm (n = 34) wide micro-gaps and on flat membranes (n = 149). The black line is the mean value for each data set.

These qualitative observations were definitively confirmed by the evaluation of the morphological parameters such as the cell orientation (Figure 5a) and aspect ratio (Figure 6a) that enable the quantification of the alignment and the elongation of the cells grown on the micro-gaps.

For the MDA-MB-231 cells grown on 2, 4, and 8 µm wide gaps, we obtained mean angle values of (2.8 ± 2.1)°, (3.5 ± 2.5)°, and (3.1 ± 2.7)°, respectively (Figure 5b). These values demonstrate that the MDA-MB-231 cells are all strongly aligned with the micro-gap direction, without significant differences among them. On the contrary, the MCF-7 cells on 2 µm, 4 µm, and 8 µm wide gaps show a weak orientation along the narrower micro-gap direction, while in presence of wider micro-gaps the effect becomes negligible, with mean angles of (10.6 ± 12)°, (18.5 ± 18)°, and (26.5 ± 24)°, respectively (Figure 5c). The strong alignment of the MDA cells with the micro-gaps, as compared to the MCF-7 cells, indicates that they have a greater ability to sense and respond to the geometrical variation and confinement in the micro-environment.

On a flat substrate, a wide distribution of the angle values was observed for both cell lines with mean angle values of (40 ± 24)° for the MDA-MB-231 and (35 ± 24)° for the MCF-7 cells. This demonstrates that the two cell lines on flat substrates exhibit a similar behavior, without assuming a preferential orientation.

The aspect ratio for MDA-MB-231 is strongly influenced by the presence of the micro-gaps and by their sizes: its mean values are (1.6 ± 0.7), (1.3 ± 0.4), and (1.0 ± 0.3) for growth on 2, 4, and 8 µm wide gaps, respectively (Figure 6b). Interestingly, the cells grown on 2 µm wide micro-gaps have a similar aspect ratio as the cells on the flat membranes (1.6 ± 0.5). On the contrary, the aspect ratio of the MCF-7 cells is not affected by the underlying structures, as shown by the aspect ratio values of (1.2 ± 0.2), (1.2 ± 0.3), and (1.3 ± 0.3) for the 2, 4, and 8 µm wide gaps, respectively. Only on the 2 μm wide micro-gap do the MCF-7 cells exhibit a significantly different aspect ratio with respect to that obtained on the flat substrate (1.3 ± 0.4).

### 3.2. Migration and Cell Density Analysis

The cell migration analysis showed that both cell lines were able to grow and migrate preferentially along the micro-gaps.

**Figure 7:**
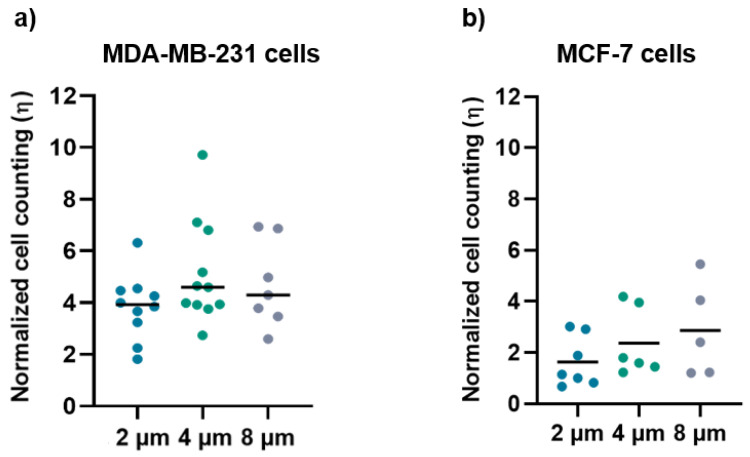
(**a**) Normalized cell counting values for the MDA-MB-231 cells on 2 µm (n = 10), 4 µm (n = 11), and 8 µm (n = 7) wide micro-gaps; (**b**) normalized cell-counting values for the MCF-7 cells on 2 µm (n = 7), 4 µm (n = 6), and 8 µm (n = 5) wide micro-gaps. The black line is the mean value for each data set.

The normalized ratio of cell density for MDA-MB-231 (Figure 7a) and MCF-7 (Figure 7b) grown on the micro-gaps and on the surrounding membrane is larger than 1 for both cell lines, regardless of the width of the micro-gaps. The mean values of cell density for the cells grown on 2 µm, 4 μm, and 8 µm are (3.8 ± 1.2), (5 ± 2), and (4.7 ± 1.6), respectively, for MDA-MB-231 cells, and (1.6 ± 1), (2.3 ± 1.3), and (2.9 ± 1.9) for MCF-7cells. These data indicate an accumulation of cells along the micro-gaps and the effect is more prominent in the case of the MDA-MB-231 cell line. Indeed, all the cells trapped in the gaps were healthy, and in a few videos, we were able to observe cell division while a given cell was lying on the gap.

**Figure 8:**
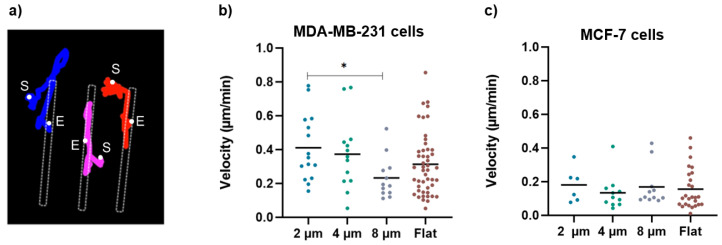
(**a**) An example of cell-tracking analysis for cells grown on 4 um wide micro-gaps to calculate the velocity of migrating cells. The colored lines indicate the trajectories obtained for different cells during migration analysis (S = start point; E = end point). (**b**) Velocity values for MDA-MB-231 cells on 2 µm (n = 15), 4 µm (n = 13), and 8 µm (n = 12) wide micro-gaps and on flat membranes (n = 48). (**c**) Velocity values for MCF-7 cells on 2 µm (n = 6), 4 µm (n = 11), and 8 µm (n = 11) wide micro-gaps and on flat membranes (n = 26). The black line is the mean value for each data set.

Consistently, the larger gaps significantly halved the migration velocity of the MDA-MB-231 cells (Figure 8b), which, on the 8 µm wide gaps, was (0.2 ± 0.1) µm/min compared to that measured on the flat substrates (0.3 ± 0.2) µm/min and that measured on the 2 µm wide gaps (0.4 ± 0.2) µm/min. Moreover, as exemplified in Figure 8a, which reports the path followed by three independent cells on a 4 µm wide gap, once a cell becomes captured by the gap, it continues to move back and forth along the gap, but very seldom returns to the flat area. This in turn explains the large values of the ratios of cell density discussed above. On the contrary, the migration velocity analysis regarding the MCF-7 cells showed no significant difference as a function of gap size. Moreover, MCF-7 cells migrate at a lower velocity than MDA-MB-231 cells on all structures, which is in line with the literature showing that MDA cells migrate faster than MCF-7 cells due to their metastatic potential [26].

### 3.3. Matrix Deposition

**Figure 9:**
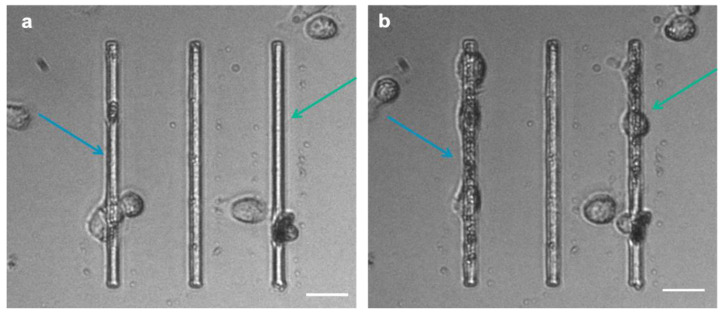
Cell-tracking frames (extracted from the SM1 videos) show MDA cancer cells grown on the micro-gaps (4 µm wide): the arrows indicate the zones where cells have produced ECM at the beginning (**a**) and 10 h after migration analysis (**b**). (Scale bar: 25 µm).

**Figure 10:**
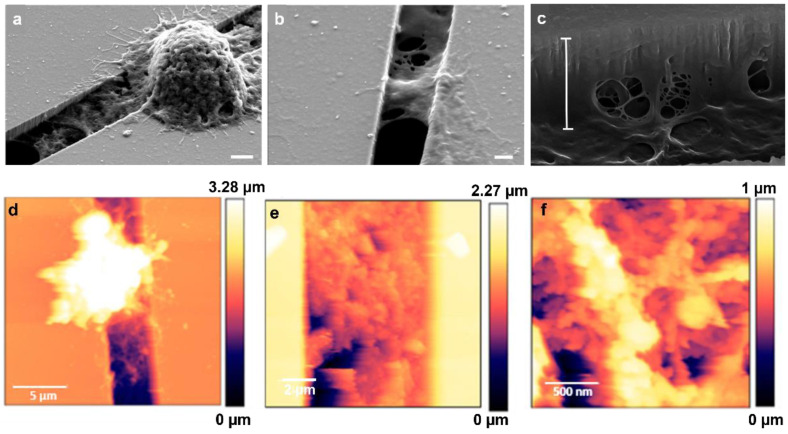
(**a**) SEM images of matrix residues under MDA-MB-231 cells adhering to a 4 µm wide gap (scale bar 2 µm); (**b**) SEM image of matrix residues on the 2 µm wide gap (scale bar: 1 µm); (**c**) SEM image with tilted view of matrix deposited inside the gaps—the measured depth of residues (white bar) is 1.8 µm (scale bar: 100 nm); (**d**) AFM image (scan: 20 µm × 20 µm) of the structures deposited on the micro-gaps (2 μm wide) by the MDA-MB-231 cells after 24 h of migration; (**e**,**f**) AFM images performed on a magnified image of the layer deposited in the gap (scans: 10 µm × 10 µm and 2 µm × 2 µm, respectively) taken across the micro-gaps (2 μm wide).

Interestingly, we observed that MCF-7 and MDA-MB-231 cells migrating on micro-gaps leave extracellular matrix (ECM)-like residues on the micro-gaps on which they move, as shown SM1 in video and Figure 9. SEM and AFM structural characterizations were performed on the fixed and dehydrated samples 24 h after migration. The SEM imaging revealed the presence of a layer corresponding to the curvature of the water meniscus and resembling the structures that constitute the extracellular matrix, as shown in Figure 10a,b, with a depth of 1.8 µm (light blue bar) (Figure 10c). AFM imaging was performed, confirming the presence of the residues around the gaps (Figure 10d–f). A higher magnification of the images revealed the presence of a matrix within the structures organized with a periodicity (60 ± 5) nm compatible with typical fibers of the ECM, such as collagen.

## 4. Discussion

### 4.1. Design and Fabrication of the 2D Micro-Pattern Assembly

In this paper, we presented the development of an assembly based on substrates patterned with micro-gaps thin enough to stop water and enable the maintenance of a water meniscus on the micro-gaps. Accordingly, we performed experiments in a so called half-wet condition enabling us to investigate the cell behavior in a region with peculiar mechanical and topological properties. The half-wetting configuration was achieved by a specially designed 2D micro-patterned assembly, which allows for the exposure of only one side of the substrates to the culture medium, due to the formation of a water meniscus across the micro-gaps. To enhance cell adhesion, the substrates were functionalized with APTES, an amino silane commonly used in biosensors [27] to create an ammine monolayer that promotes a specific cell’s adhesion [28], using a CVD-based protocol, as reported in the literature [24,25].

The chosen dimensions of our patterns allowed us to explore the “nuclear limit” and the spatial confinement above which cell migration requires matrix remodeling due to the stiffness of the nucleus [5,29], both for MDA and MCF-7 cells.

### 4.2. Cell Morphology as a Function of the Micro-Gap Size

The morphological evaluations confirmed by the quantification of the aspect ratio and the cell orientation showed that MDA-MB-231, similar to invasive breast cancer cells, respond to the presence of surface topographies and can align and adapt their morphology accordingly. More specifically, the elongation of cells adhering to 2 µm wide gaps or flat Si_3_N_4_ membranes gradually decreases with an increasing gap size so that cells on 4 and 8 μm wide gaps exhibit an increasingly more rounded shape. In general, the effect of topography on cell shape can be explained by the phenomenon of contact guidance, according to which the surface topography affects the morphology and the behavior of the cells, leading to their alignment and increasing their migration rate on fibril-like structures [4,30,31]. In our configuration, the presence of a water meniscus may play an important role as it can create a three-dimensional and confined environment with a lower stiffness and a smaller number of sites for cell adhesion. For wider gaps, the water meniscus likely creates a larger area with a lower surface tension that cells may perceive as a softer substrate, causing them to change their morphology toward a rounder appearance. This could be consistent with the morphological change of MDA-MB-231 cells, which pass from an elongated to a rounded shape when cultured on soft substrates [32], but could also be explained by the fact that cells loosely adherent to confined environments may exhibit an amoeboid and rounded morphology [5]. The non-invasive MCF-7 breast cancer cells were able to self-align when grown on the micro-gaps but showed less variability in morphology compared with the MDA-MB-231 cells. These results are consistent with the literature suggesting that non-invasive cells such as MCF-7 have a lower ability to perceive the effects of topographical features compared to their aggressive counterparts [33].

Overall, these results suggest that topographic conditions differentially affect the aspect ratio of MDA-MB-231 and MCF-7 cells. The MDA-MB-231 cells grown on the 4 and 8 μm wide gaps can perceive the surface tension of the meniscus and adapt to a different physical confinement effect by assuming a rounded morphology, which resembles the mesenchymal-ameboid transition during metastatic processes [34]. As shown, MCF-7 are also more elongated on flat substrates than on micro-gaps, but with a smaller impact than in MDA-MB-231 cells. So, the lower invasiveness of MCF-7 cells is reflected in their lower responsivity to topographical changes, as shown in the literature [33]. However, to better characterize the connections between tumoral aggressivity and substrate responsivity, further experiments with different cell lines would be required.

### 4.3. Cell Migration in Micro-Gaps

The single-cell tracking analysis performed on the MDA-MB-231 cells during their migration in the 2D micro-patterned substrates showed that these cells preferentially adhere to the micro-gaps rather than the surrounding flat membrane and can migrate along them at velocity values that vary as a function of the gap size. While the cells on narrow (2 µm) micro-gaps migrate at the same velocity as the cells adhering to the flat Si_3_N_4_ membrane, they reduce their migratory rate on larger (4 and 8 µm) micro-gaps. This can be explained by considering that the width of the gaps influences the extent of the underlying meniscus, giving rise to water–air interfaces that can be perceived differently by the cells. It is likely that the water meniscus acts as a physical confinement through surface tension, reducing the velocity of the cells when its size expands. A second explanation could be that there are no solid anchorage points on the meniscus; thus, the cells’ progression is slowed down because of the reduction in pulling force. On the other hand, it is interesting to notice that the cells seem to remain confined inside the gaps, although the anchoring possibilities would be much higher outside.

The lower velocity values of the MCF-7 cells compared to the MDA-MB-231 cells are consistent with the available literature [35] and can be explained by the demonstrated high metastatic potential of MDA-MB-231 cells [26].

Remarkably, in our experiments, the cells migrated back and forth along the patterns without a preferential course, unlike the case of the physiological condition. There, biomolecular cues and concentration gradients drive the directionality and speed of cell migration. We purposefully did not use a chemoattractant or chemo-blocker because we aimed at investigating the cellular responses to only the physical features. Therefore, the absolute values of speed and morphology may not be consistent with what happens in a real biological system. However, despite the important simplifications adopted, we were able to describe in detail the different behaviors associated with two different degrees of invasiveness.

An interesting aspect of cell migration along the micro-gap is the deposition of a matrix-like structure. The matrix deposition along the micro-gap suggests that its secretion is functional towards cell adhesion to and migration in the meniscus regions. This is extremely relevant considering that the ECM has been shown to regulate the morphology and the invasive behavior of cancer cells [36]. In this context, determining the role of the surrounding matrix and modulating its properties could be extremely useful for gaining an in-depth understanding of the tumor-spreading process for the development of novel and targeted therapies against it [37].

## 5. Conclusions

We have developed a novel 2D-micropatterned system, perfectly compatible with standard optical microscopy, which, thanks to the presence of micro-gaps with different widths, allows us to investigate the effects of hydrophilic/hydrophobic interfaces, water menisci, and surface curvature on cancer cell behavior (i.e., cell morphology, orientation, migration, etc.).

Our experimental results show that the highly invasive MDA-MB-231 cancer cells respond remarkably to the structures, while the less invasive MCF7 cells are less affected by them.

The 2D micro-patterned system presented in this work uses a water–air interface that can be tuned to mimic some features of the tumor microenvironment, with different topological and mechanical properties. This unique configuration enables the easy analysis of cell morphology and migration using conventional imaging techniques.

The study of cell migration can be time consuming and laborious due to its intrinsically complex nature and since the technical limitations of assays’ development involve multiple factors that can influence migration.

The 2D-microstructured system was used to investigate the effects of only physical cues, but chemical cues can be introduced to investigate the combined effect of topology and chemistry on cell migration. Moreover, differently shaped micro-gaps, which enable the identification of specific mechanotransduction pathways, can also be implemented. Finally, the use of drugs interfering with cell migration can help to better understand the performance of 2D systems for clinical application.

Although a 2D-micro-structured system still needs to be optimized and integrated into automated analysis, it could have promising and relevant implications not only for oncology research but also for all other fields where cell migration may play a crucial role, such as tissue engineering and inflammation-related research.

## Supplementary Materials

The following supporting information can be downloaded at: https://www.mdpi.com/article/10.3390/bios12100822/s1. Figure S1: (a) an example of KLayout scheme showing the final pattern of microstructured gaps; (b) SEM image of the array with microstructured gaps (8 μm width) on the Si_3_N_4_ membrane. Figure S2: (a) Schematic diagram of the of the assembly used as chamber of measurement for the experiments with living cells. Each chip was glued to the bottom of a Petri dish on which 4 holes were created, one chip for each hole. (b) Pictures of the assembly (top and side view) after mounting of chips and filling with culture medium: commercial Petri dish with homemade holes, PMMA ring spacer, 2 commercial caps of Petri dish. Figure S3: Evaluation of APTES functionalization efficacy. (a) Contact angle measurements to verify the functionalization and assess its stability after 24 hours; (b) Measurements of the average cell density (cells/cm^2^) show the adhesion-enhancing properties of the APTES. Figure S4: An example of image where active count area (CA), in blue, and gap area (GA), in green, are drawn. The number 1 and 2 indicate two cells counted inside the gap area and count area, respectively.
