# Supplementary material for: Water–Air Interface to Mimic In Vitro Tumoral Cell Migration in Complex Micro-Environments
Source: Biosensors (Basel). 2022 Oct 3;12(10):822. doi: 10.3390/bios12100822 (PMC9599853; doi:10.3390/bios12100822)
Supplement: Supplementary file 1 [file biosensors-12-00822-s001.zip › biosensors-1908890-supplementary materials/biosensors-1908890-supplementary-forXML/biosensors-1908890-Supplementary.pdf]

Supplementary material

Design and fabrication

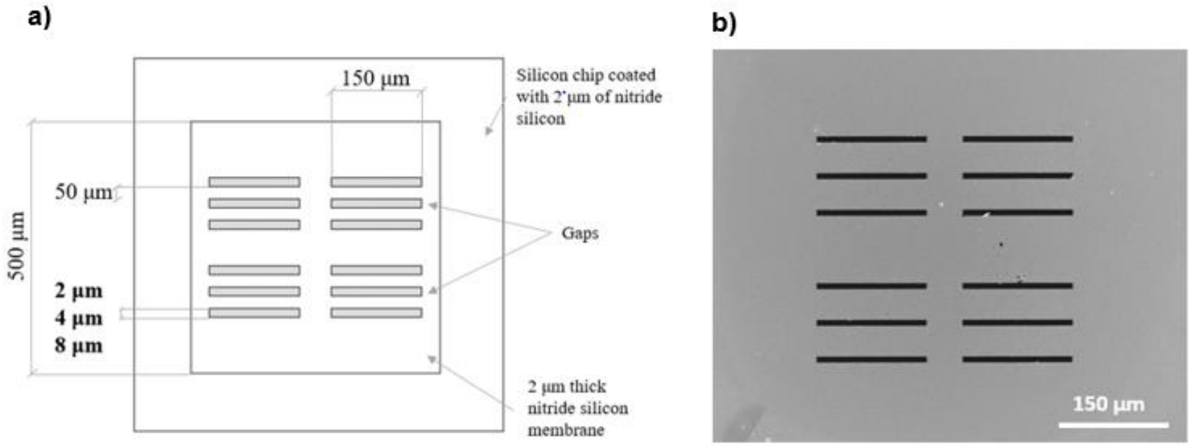

Figure S 1 : (a) an example of KLayout scheme showing the final pattern of microstructured gaps; (b) SEM image of the array with microstructured gaps (8 um width) on the Si_3N_4 membrane.

Half-wet assembly

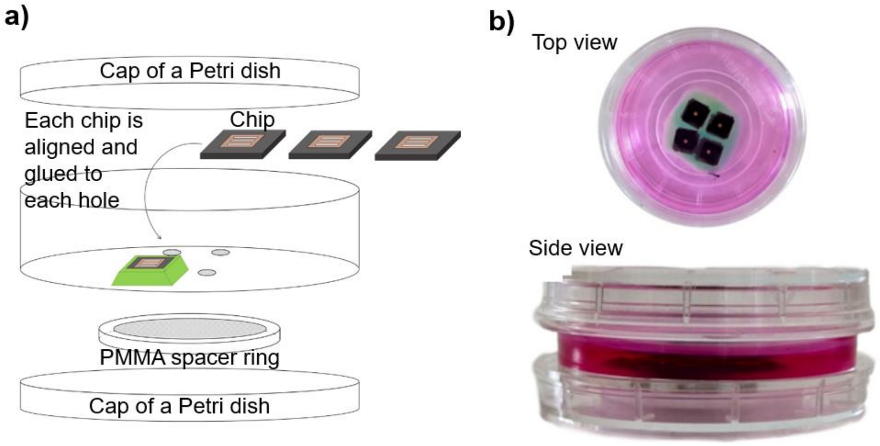

Figure S2: a) Schematic diagram of the of the assembly used as chamber of measurement for the experiments with living cells. Each chip was glued to the bottom of a Petri dish on which 4 holes were created, one chip for each hole. b) Pictures of the assembly (top and side view) after mounting of chips and filling with culture medium: commercial Petri dish with homemade holes, PMMA ring spacer, 2 commercial caps of Petri dish.

Functionalization

S3 a – Contact angle measurements

Functionalization was verified with contact angle (CA) measurements performed with a DataPhysics optical instrument OCA 15Pro (DataPhysics Instruments GmbH, Germany) at room temperature, by placing 2 μL of Milli-Q water on the substrates before and after the functionalization process. Measurements were repeated 24 hours after the functionalization to assess the APTES stability. CA values were determined averaging four position on three samples for each condition.

S3 b– Efficacy of the APTES functionalization

The efficacy of the APTES functionalization for the cell-adhesion purposes was verified measuring the cell density (cells/cm^2) on native and functionalized surfaces. Cells were seeded onto substrates following the protocol described in the "Cell seeding protocol" section. Bright-field images of the samples were then analyzed with the "Cell Counter" plug-in of Image J. Statistical analysis was performed using GraphPad Prism (version 8.4.3) and significance between cell densities on the differently functionalized substrates was assessed by a two-tailed Student's t test, where $P \leq 0.05$ was considered statistically significant (**).

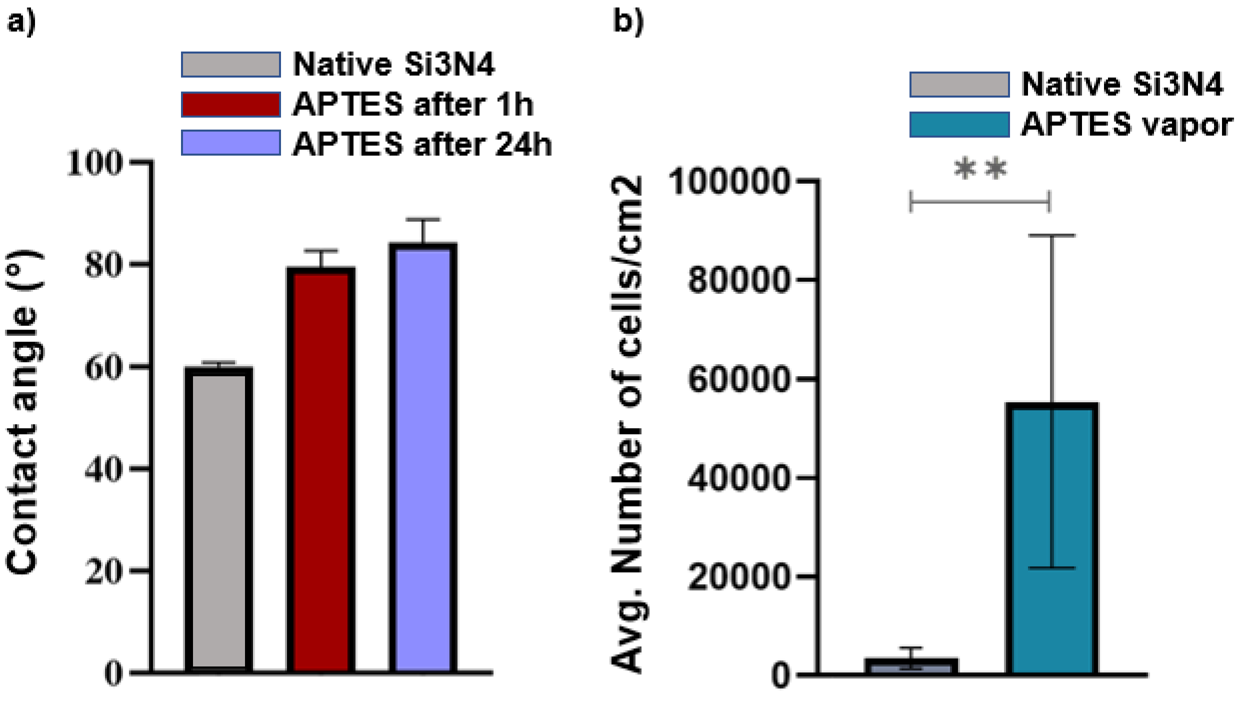

Figure S3: *Evaluation of APTES functionalization efficacy. a) Contact angle measurements to verify the functionalization and assess its stability after 24 hours; b) Measurements of the average cell density (cells/cm^2) show the adhesion-enhancing properties of the APTES.*

The functionalization of the substrates with APTES was verified measuring the contact angle. Consistently with available literature [16], the APTES functionalization led to an increase in the

contact angle values, from (60±0.7)° for the native Si_3N_4 surface and (79 ±3)° one hour after the APTES coating, as shown in Figure S3 a. Moreover, the reduced variation of the contact angle after 24 hours it is indicative of a stable functionalization.
The evaluation of the adhesion-enhancing properties of the APTES was based on the quantification of the cell density on native and functionalized Si_3N_4 membranes. As shown in Fig. S3 b, cell density increases when the substrates are functionalized with APTES.

S4 – Cell density evaluation

The quantification of cells adhering to the micro-gaps was done by calculating the normalized ratio of cells grown on the gaps (η). This parameter considers the number of cells that are somehow in contact with the gap to the whole number of cells on the *active count area* (*CA*).
The *active count area* is defined as the area that a single cell could explore during migration. To quantify this value, the migration tracking analysis was performed on MDA-MB-231 and MCF-7 cancer cells by following them for 24h. For each chip, an image was acquired in bright field (BF) microscopy and the whole distance reached by a single cell was calculated with the "Manual Tracking" plug-in of the ImageJ software. By this distance it is possible to obtain the value of the area covered by a cell that is around $2x10^5\ \mu m^2$ (indicated in blue in the Figure 8a) for both cell lines.
In this way the cells that adhere too far from the gap pattern have been excluded from the analysis. In addition, the gap area (GA) was defined inside the active count area, as the area around each gap that includes the cells that are in contact with it. Once defined CA and GA, the count of cells on that area was calculated as follow.

Cell counting was performed using the "Cell Counter" plug-in of ImageJ.
Normalization of the cell counting was achieved with the following parameter: (η):

$$\eta = \frac{n_{cells\ gap}}{\sum GA} : \frac{N_{cells\ outside\ gap}}{CA - \sum GA}$$

where $n_{cells\ gap}$ are the cells in the gap area, shown in green in the left image; $N_{cells\ outside\ gap}$ are the cells outside the gap area but inside the active count area; $\sum GA$ is the sum of the GA calculated for each chip (containing 12 gaps, as described in the paragraph above).

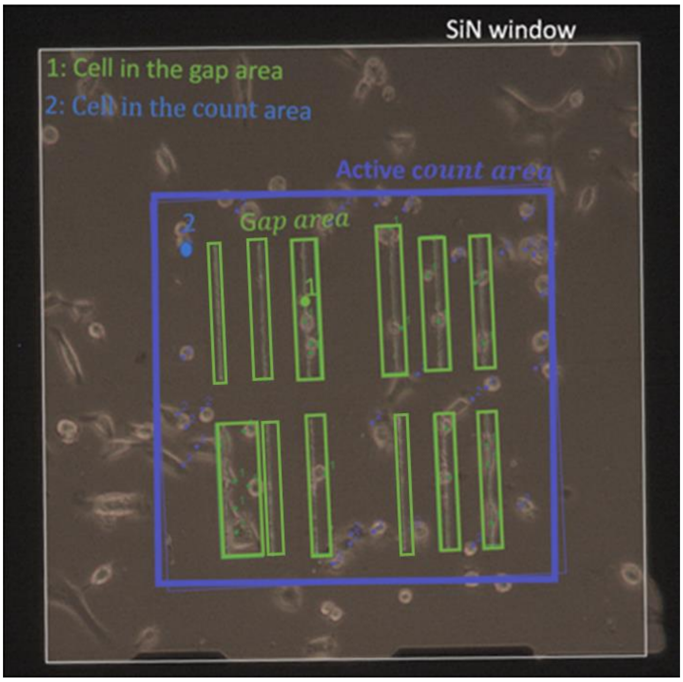

Figure S4: *An example of image where active count area (CA), in blue, and gap area (GA), in green, are drawn. The number 1and 2 indicate two cells counted inside the gap area and count area, respectively.*

SM1 – ECM deposition during MDA cells migration on micro-gaps

The movie shows MDA-MB-231 cells migrating on micro-gaps (4 µm wide) that leave extracellular matrix (ECM)-like residues on the micro-gaps on which they move. Images were acquired using the Olympus Scan R 3.1 microscope, equipped with the live-cell imaging module. In time lapse an image with 100 ms of exposure time was acquired every 15 minutes for a total time of 10 hours.
